# Effects of aqueous ozone treatment on microbial growth, quality, and pesticide residue of fresh‐cut cabbage

**DOI:** 10.1002/fsn3.1870

**Published:** 2020-12-16

**Authors:** Chenghui Liu, Chen Chen, Aili Jiang, Yanhui Zhang, Qiqi Zhao, Wenzhong Hu

**Affiliations:** ^1^ College of Life Science Dalian Minzu University Dalian China; ^2^ Key Laboratory of Biotechnology and Bioresources Utilization (Dalian Minzu University) Ministry of Education Dalian China

**Keywords:** aqueous ozone, cabbage, disinfection, pesticide residue, produce quality

## Abstract

The influence of aqueous ozone (1.4 mg/L) treatment for 1, 5, and 10 min on the microbial growth and quality attributes of fresh‐cut cabbage during storage at 4°C for 12 days was evaluated. The pesticide residue removal effect of aqueous ozone treatment for 5 min was also determined. The results show that the growth rates of aerobic bacteria, coliforms, and yeasts were significantly inhibited (*p* < .05) by aqueous ozone treatment during storage; treatment for 10 min showed the greatest inactivation of bacteria, coliforms, and molds. Aqueous ozone stimulated initial respiratory metabolism compared with that of the control. Aqueous ozone treatments reduced ethylene production and improved the overall quality of fresh‐cut cabbage. In addition, the effect of aqueous ozone treatment for 5 min on the removal of trichlorfon, chlorpyrifos, methomyl, dichlorvos, and omethoate from fresh‐cut cabbage was greater (*p* < .05) than that of the control. These results indicate that aqueous ozone treatment for 5 min could be an economic and effective method to remove pesticide residues and enhance the storability of fresh‐cut cabbage.

## Graphical abstract

Aqueous ozone treatment inhibited microbial counts on fresh‐cut cabbage. Aqueous ozone inhibited ethylene production and extended the shelf life of cabbage aqueous ozone treatment for 5 min is particularly suitable for fresh‐cut cabbage.

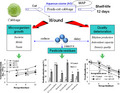

## 1 INTRODUCTION

Fresh‐cut fruits and vegetables have increased in popularity owing to their health benefits, freshness, and convenience, stimulating new research in this area (Baselice, Colantuoni, Lass, Nardone, & Stasi, 2017). Cut processing has negative effects on product quality; it can result in microbial infection, off‐flavor development, cut‐surface browning, and textural breakdown (Ma, Zhang, Bhandari, & Gao, 2017). In particular, cut processing causes damage to the original cells and releases cellular nutrients at the wound site, which not only makes fresh‐cut fruits and vegetables more susceptible to microbial infection, but also provides nutrition for microbial growth. In addition, the potential health hazard of pesticide residues in fresh‐cut products has also attracted people's attention. Accordingly, it is crucial to control microbial growth and ensure the safety of fresh‐cut produce during processing and subsequent storage.

Washing with chemical disinfectants is a critical step to reduce microorganisms and pesticide residues, which are detrimental to the quality of fresh‐cut fruits and vegetables (Ali, Yeoh, Forney, & Siddiqui, 2018; Haute, Tryland, Escudero, Vanneste, & Sampers, 2017). Among the available chemical disinfectants, chlorine is the most widely used in the fresh‐cut produce industry owing to its low cost, convenience, and effectiveness (Chen & Hung, 2016; Luo et al., 2018). However, chlorine and chlorine‐associated compounds are prohibited in some European countries due to the carcinogenicity of chlorinated by‐products (Meireles, Giaouris, & Simões, 2016). Therefore, the development of alternative disinfection methods is a challenge for both academia and industry. As a strong oxidant, aqueous ozone has high disinfection efficacy at low concentrations and short contact times (Botondi, Moscetti, & Massantini, 2016). In addition, it rapidly decomposes into oxygen and does not leave a residue in treated products (Miller, Silva, & Brandão, 2013). Accordingly, washing with aqueous ozone is a highly efficient and safe method for the preservation of fresh‐cut fruits and vegetables and can be a good substitute for washing with chlorine. A study conducted by Silveira, Oyarzún, and Escalona (2018) reported that fresh‐cut grape berries sanitized with aqueous ozone showed similar microbial counts, high total polyphenol contents, and antioxidant capacity compared with NaOCl used by the industry. Wang et al. (2019) demonstrated that lactic acid plus aqueous ozone led to the greatest reductions in microbes and did not cause additional quality loss compared with chlorine treatment. By comparing some research results, it was found that aqueous ozone can achieve similar reduction of microorganisms in fresh‐cut produce as electrolyzed water and hydrogen peroxide (Ali et al., 2018). Although aqueous ozone can lead to some safer fresh‐cut fruits and vegetables with an extensive shelf life, the overall quality of the final products, in terms of physico‐chemical, sensorial, and nutritional features, must be ensured. Several researchers evaluated the effects of aqueous ozone on the quality of some fresh‐cut fruits and vegetables (Liu, Tao Ma, & W. Z., Tian M. X., & Sun, L., 2016; Papachristodoulou, Koukounaras, Siomos, Liakou, & Gerasopoulos, 2018). As occurs in produce decontamination, the aqueous ozone effects on quality are also highly influenced by product attributes (Miller et al., 2013). For each fresh‐cut product, there is a threshold of time and concentration for aqueous ozone treatment above, which it negatively impacts the quality of the treated sample (Chen et al., 2019). In addition, some research results have shown that aqueous ozone can effectively remove several pesticides from fruits, vegetables, and their fresh‐cut products (Camara, Barba, Cermeño, Martinez, & Oliva, 2017; Lozowicka, Jankowska, Hrynko, & Kaczynski, 2016; Souza et al., 2018).

Cabbage (*Brassica oleracea* L.) is an economically important vegetable due to its nutritional composition, versatility, and the potential for industrial processing. Fresh‐cut cabbage has become a salable product in restaurants, dining commons, and fast food outlets, with growing market demand (Ranjitha, Rao, Shivashankara, & Roy, 2018; Sow, Tirtawinata, Yang, Shao, & Wang, 2017). In contrast to intact cabbage, fresh‐cut cabbage has a limited shelf life as a result of rotting, nutrient loss, surface blackening, off odors, pesticide residues, and other factors (Sow et al., 2017). Thus, it is necessary to develop a washing technology that can be applied to fresh‐cut cabbage to reduce microbial contamination, improve product quality, and remove pesticide residues. There is no report so far on the application of aqueous ozone for preservation on fresh‐cut cabbage. The objective of the present study was to examine the effects of washing with low‐concentration aqueous ozone (containing ozone 1.4 mg/L) for 1, 5, and 10 min on the microbial load and main quality parameters of fresh‐cut cabbage and evaluate the removal efficiency of six pesticide residues using aqueous ozone treatment for 5 min.

## 2 MATERIALS AND METHODS

### 2.1 Raw materials and chemicals

Cabbages (*Brassica oleracea* var. capitata L.) were purchased from a local farm in Dalian City, P.R. China, transported to the laboratory. Cabbages with similar weights (500 ± 20 g) and sizes, and absence of wounds were selected.

Plate count agar, violet red bile agar, potato dextrose agar, and peptone were purchased from Aoboxin (Beijing, China). The analytical grade methanol, sodium carbonate, HCl, gallic acid, sodium chloride, acetonitrile, and toluene were obtained from Kemiou (Tianjin, China). The FRAP (Ferric ion reducing antioxidant power) reagent was supplied from Comin (Suzhou, China). Folin–Ciocalteu reagents were purchased from Solarbio (Beijing, China). Commercial pesticides were used to incubate cabbage, including chlorpyrifos (45% purity, Jiahui Chemical Co., Ltd., Suzhou, China), β‐cypermethrin (4.5% purity, Shandong Dacheng Biochemical Co., Ltd., Zibo, China), trichlorfon (90% purity, Shandong Dacheng Biochemical Co., Ltd., Zibo, China), methomyl (90% purity, Longdeng Chemical Co., Ltd., Jiangsu, China), dichlorvos (77.5% purity, Shandong Dacheng Biochemical Co., Ltd., Zibo, China), and omethoate (40% purity, Beijing‐Tianjin Pesticide Co., Ltd., Tianjin, China). Analytical standards (>99% purity) of chlorpyrifos, β‐cypermethrin, trichlorfon, methomyl, dichlorvos, and omethoate were supplied by Dr. Ehrenstorfer (Augsburg, Germany). LC‐MS grade acetonitrile, formic acid, and chromatography grade water were acquired from Merck (Darmstadt, Germany).

### 2.2 Aqueous ozone preparation and sample treatment

Aqueous ozone with ozone concentration of 1.4 mg/L was prepared by an ozone generator (HY‐SL; YingLong electronics Co., LTD, Fujian, China) according to the method reported by Liu et al. (2016). Bubbling ozone gas was pumped into sterile deionised water at a controlled flow rate of 800 mg/hr for 30 min to get a saturated ozone concentration at 4 ± 1°C. The concentration of ozone in aqueous ozone was determined by the official indigo colorimetric method (Bader & Hoigné, 1981). The aqueous ozone was used immediately after the ozone concentration stably reached 1.4 m/L.

The outermost damaged and diseased leaves of cabbages were stripped, and plants were washed with tap water for 5 min and dried naturally. Subsequently, root segments and cores were cutoff, and then leaves were cut into slices of about 1 × 5 cm with sanitized sharp stainless steel knives. The slices were dispersed to prevent adhesion and then randomly divided into three aqueous ozone treatment groups and a control group. For the three aqueous ozone treatment groups, fresh‐cut cabbages were washed in continuous flow aqueous ozone for 1, 5, and 10 min at 4°C, designated as AO‐1, AO‐5, and AO‐10, respectively. The control group was treated with distilled water instead of aqueous ozone for 5 min. The ratio of washing solutions and samples was 3:1 in each case. After each treatment, about 80 g of the fresh‐cut cabbage samples was placed on sterile polyethylene trays, taken into a biosafety cabinet to let them dry, packed with food‐grade polyethylene cling film (10 μm thickness, permeability characteristics: O_2_ = 10 030 cm^3^ m^‐2^ 24 hr^‐1^ bar^‐1^; CO_2_ = 36 300 cm^3^ m^‐2^ 24 hr^‐1^ bar^‐1^), and stored at 4 ± 1°C and 90% relative humidity for 12 days. Samples were obtained for evaluation at days 0, 4, 8, and 12.

### 2.3 Microbiological measurements

Counts of total aerobic bacteria, coliforms, yeasts, and molds were assessed according to the Chinese GB standards (GB4789.2–2016; GB4789.3–2016; GB4789.15–2016). In brief, the samples (25.0 g) were blended with 225 ml of sterile 0.1% peptone water for 2 min in a sterile homogeneous bag using a sterile homogenizer (Scientz‐09; Ningbo Scientz Biotechnology Co. Ltd., Ningbo, China). To examine the counts of bacteria, coliforms, mold, and yeast, 1 ml was aseptically pipetted from each diluted sample and placed into the appropriate culture media: plate count agar medium for total aerobic bacteria incubated at 36 ± 1°C for 48 ± 2 hr; violet red bile agar for coliforms incubated at 36 ± 1°C for 48 ± 2 hr; and potato dextrose agar with chloramphenicol for yeasts and molds incubated at 28 ± 1°C for 3–5 days. For coliforms, only dark red colonies were counted. Molds were identified by the examination of colony and cell morphology. All microbial counts were carried out in triplicate and expressed as log_10_ CFU/g of fresh weight.

### 2.4 Respiration rate and ethylene production

Samples (55 g) were placed in 550 ml sealed containers at 4 ± 1°C. After standing for 1 hr, the concentrations of CO_2_ and C_2_H_4_ in the headspace gas were evaluated using a gas analyzer (F‐940; Felix, Kyoto, USA) at 4 ± 1°C. The respiration rate and ethylene production were evaluated in triplicate and calculated as mg CO_2_ kg^‐1^ hr^‐1^ and µl C_2_H_4_ kg^‐1^ hr^‐1^ (Liu et al., 2016).

### 2.5 Quality attributes

#### 2.5.1 Weight loss

The weights of treated and untreated samples during storage were determined using a balance (AL204; Mettler‐Toledo Group, Zurich, Switzerland) with a precision of 0.1 mg. The weight loss was carried out in triplicate and expressed as a proportion of the initial fresh weight (%).

#### 2.5.2 Total phenol content

Total phenol content was evaluated, following the procedure of Singleton, Orthofer, and Lamuela‐Raventós (1999) with slight modification. Samples of 5 g were homogenized with 20 ml of cold methanol using an Ultra‐Turrax homogenizer (IKA Works, Inc., Wilmington, NC, USA). Homogenates were centrifuged (TG‐20M; Cence Laboratory Instrument Development Co., Ltd., Hunan, China) at 23,120 × *g* for 20 min at 4°C. Sample aliquots of 2 ml were taken from the clear supernatant, mixed with 2 ml Folin–Ciocalteu reagents followed by 20 ml of sodium carbonate (0.075 g/ml), then diluted to 50 ml with distilled water, and incubated for 90 min at room temperature, and the absorbance was read at 750 nm. Gallic acid was used as a standard. The total phenol content was carried out in triplicate and expressed as g gallic acid equivalent (GAE) in 1 kg of fresh‐cut cabbage fresh weight (g/kg FW).

#### 2.5.3 Antioxidant capacity assay

The antioxidant capacity of fresh‐cut cabbages was investigated using a FRAP assay, which was carried out following the methods of Alothman, Kaur, Fazilah, Bhat, and Karim (2010) modified to fit a 96‐well plate. The extraction method was the same as that of total phenol. 24 μl of diluted sample methanol extract was mixed with 180 μl of FRAP reagent, and the reaction was conducted for 20 min at 37°C. The absorbance of the mixture was measured at 593 nm using a Multiskan GO (1,510, Thermo Scientific, Vantaa, Finland). The FRAP reagent was prewarmed at 37°C and was always freshly prepared by mixing 2.5 ml of a 10 mM 2,4,6‐tris (1‐pyridyl)‐5‐triazine (TPTZ) solution in 40 mM HCl with 2.5 ml of 20 mM FeCl_3_.6H_2_O and 25 ml of 0.3 M acetate buffer pH 3.6. The antioxidant activity of fresh‐cut cabbages was evaluated in triplicate and expressed as µmol Fe^2+^‐TPTZ g^‐1^ FW.

#### 2.5.4 Color measurement and sensory evaluation

The visual color characteristics of fresh‐cut cabbages were measured using a Minolta Chromameter CR‐400 (Minolta Corp., Osaka, Japan). The results were obtained on the basis of CIELAB (L*, a*, b*) color space. L* defines the lightness, while a* and b* define the red‐greenness and blue‐yellowness, respectively. The browning index (BI) was used as an indicator of brown color intensity and shown in following equation: BI = 100 (×−0.31)/0.172; where x = (a* + 1.75 L*)/(5.645 L* + a*−3.012 b*) (Olivas, Mattinson, & Barbosa‐Cánovas, 2007). Ten slices of cabbages were randomly selected from each group, and two sites per slice were analyzed.

The overall quality was assessed by a panel of eight trained individuals (four men and four women; aged 25–55 years) based on the visual appearance, aroma, taste, and texture of the fresh‐cut cabbage. Evaluations were scored on a nine‐point scale, simply described as follows: 1 = extremely poor, 3 = poor (just below the limit of marketability, 5 = acceptable (limit of marketability), 7 = good, and 9 = excellent. The overall appreciation of a sample was measured on the same scale and is referred to as the overall quality score (Aguayo, Escalona, Silveira, & Artés, 2014).

### 2.6 Residual pesticide measurement

#### 2.6.1 Inoculation of cabbages with pesticides

To inoculate cabbages with pesticides, 2.5 L of working solution was prepared by mixing 10 mg/L chlorpyrifos, 10 mg/L β‐cypermethrin, 1 mg/L trichlorfon, 2 mg/L methomyl, 5 mg/L dichlorvos, and 0.2 mg/L omethoate. These pesticides used for inoculation were commercial products. Then, 0.5 kg of whole cabbage leaves were soaked in the working solution for 10 min. Samples were air‐dried for 3 hr in a fume hood at 20°C and maintained in a refrigerator for 12 hr at 4°C to allow for pesticide attachment.

#### 2.6.2 Sample treatment

Cabbages inoculated with pesticides were cut into 1 × 5 cm slices and randomly divided into three groups. One group was used to determine the initial concentration of pesticides on fresh‐cut cabbage, another group was treated with 1.4 mg/L aqueous ozone for 5 min (AO‐5), and the other group was treated with distilled water for 5 min (control). The ratio of washing solutions and fresh‐cut cabbages was 3:1 in each case.

#### 2.6.3 Pesticide analysis

Pesticide analysis was performed according to the procedures of the National Standard of the People's Republic of China GB 23,200.8–2016 for the determination of chlorpyrifos and β‐cypermethrin, and GB/T 20769–2008 for the determination of trichlorfon, methomyl, dichlorvos, and omethoate. The sample (40 g) was homogenized with 80 ml acetonitrile for 1 min, and 10 g sodium chloride was added and then homogenized for 1 min. The homogenate was centrifuged at 1509.3 ×   g for 5 min, and 40 ml of supernatant was collected. For chlorpyrifos and β‐cypermethrin, the Envi‐18 column (Supelco, Bellefonte, PA, USA) was prewashed with 10 ml acetonitrile before sample loading, and then the supernatant (20 ml) was purified on this column with 15 ml acetonitrile. The elution was evaporated to about 1 ml by a rotary evaporator at 40°C and then was transferred with a syringe. After that, it was filtered through 0.45 μm filter (Millipore Millex‐HN Nylon, Billerica, MA, USA) and used to gas chromatography‐mass spectrometry (GC‐MS) analysis. For trichlorfon, methomyl, dichlorvos, and omethoate, the supernatant (20 ml) was concentrated to 1 ml by a rotary evaporator at 40°C. The Sep‐Pak Vac column (Waters Corp., Milford, MA, USA) was prewashed with 4 ml acetonitrile‐toluene (volume ratio of 3:1) before sample loading, and then the concentrate sample was purified on this column with 4 ml acetonitrile‐toluene. The concentration and purification operations were repeated three times. All elution was evaporated to about 1 ml by a rotary evaporator at 40°C and then was transferred with a syringe. After that, it was filtered through 0.45 μm filter (Millipore Millex‐HN Nylon, Billerica, MA, USA) and used to liquid chromatography–mass spectrometry (LC‐MS) analysis.

GC–MS analyses were performed using Agilent 7890A/5975C GC‐MS equipment (Agilent Technologies, Palo Alto, CA, USA) equipped with an Agilent HP‐5MS column (30 m × 0.25 mm × 0.25 µm). The injection volume was 1 μl. The GC‐MS was operated in full scan mode using an ionization energy of 70 eV. The injector, interface, and ion source temperatures were 260, 280, and 230°C, respectively. The oven temperature was programmed as follows: 70°C hold for 2 min, rise to 200°C (3°C/min), to 280°C (8°C/min), and hold for 5 min. LC–MS analyses were performed using a Waters Ι class‐TQ‐S micro (Waters Co., Cork, Ireland) equipped with a Waters ACQUITY UPLC BEH C18 column (2.1 × 100 mm, 1.7 μm). The column oven temperature was maintained at 40°C, and the sample volume injected was 2 μl. A gradient elution program at 0.45 ml/min flow, in which both reservoirs contained 0.1% formic acid in (A) water and (B) acetonitrile, was used as follows: gradient 0–2 min 98% A, 2–11.5 min 2% A, 11.5–13 min 2% A, and 13.1–15 min 98% A. Detection by MS was carried out in MRM (multiple reaction monitoring), and the source used was ESI (electrospray ionization) in positive mode. Ionization voltage was 5,000 V and temperature was 500°C.

The removal efficiency of pesticides was calculated according to the following equation:

Reduction rate (%) = [(Co − Ct)/Co × 100.

where Co is the initial concentration of the pesticide on fresh‐cut cabbage and Ct represents the final concentration of the pesticide on fresh‐cut cabbage after treatment. The residual pesticides were measure in triplicate for each group.

### 2.7 Statistical analysis

The results were evaluated by analysis of variance (ANOVA) with Duncan's multiple range tests (*p* < .05) using SPSS (version 19.0; SPSS, Chicago, IL, USA).

## 3 RESULTS AND DISCUSSION

### 3.1 Microbiological analyses

**FIGURE 1:**
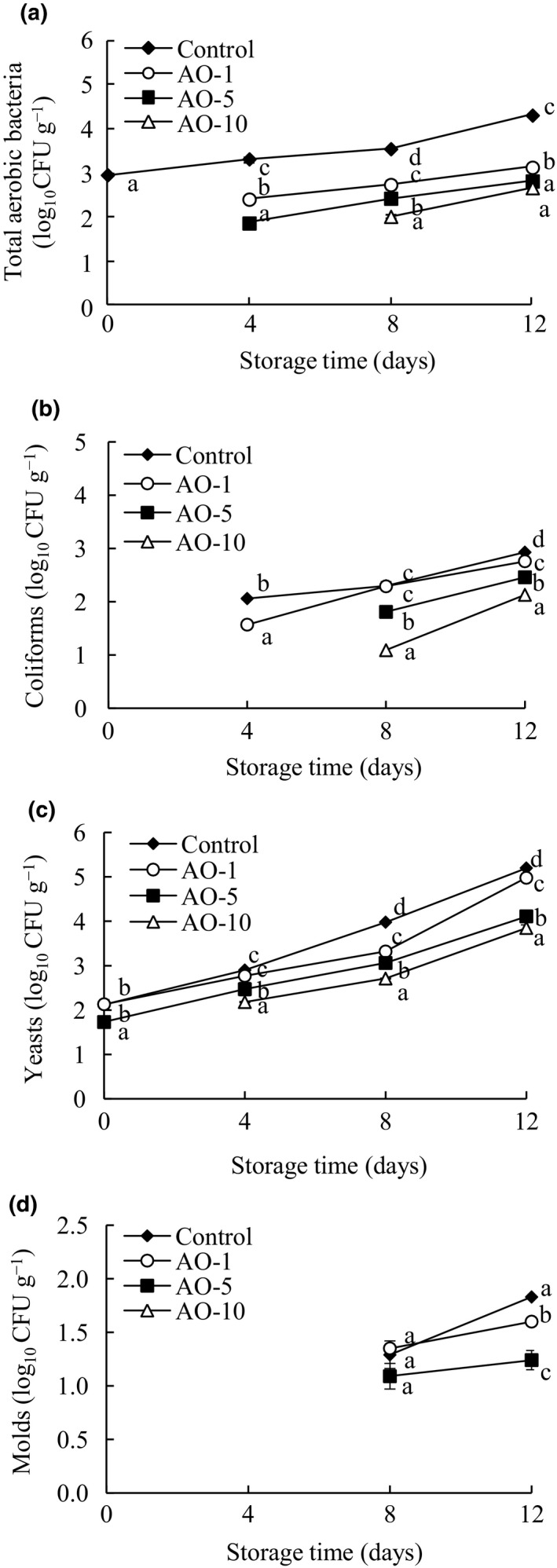
Aqueous ozone inactivation efficacy against bacteria (A), coliforms (B), yeasts (C), and molds (D) on fresh‐cut cabbages during 12 days of storage (◆, Control; ○, AO‐1; ■, AO‐5; △, AO‐10). Data shown are the means (*n* = 3) ± standard deviation. For each treatment, different lower letters show significantly different (*p *< .05) in the same storage days

As shown in Figure 1, the aerobic bacteria, coliform, yeast, and mold counts for all groups gradually increased as the storage time increased, but aqueous ozone treatment inhibited (*p* < .05) their growth during 12 days of storage. The initial total count of aerobic bacteria in the control group was 3.0 log_10_ CFU/g on day 0 and increased to 4.3 log_10_ CFU/g on day 12 (Figure 1a). However, the initial total counts of aerobic bacteria were undetectable (<1 log_10_ CFU/g), and the final total counts were reduced by 1.2, 1.5, and 1.6 log_10_ CFU/g in samples treated with aqueous ozone for 1, 5, and 10 min compared with levels in the control. As shown in Figure 1b, the coliform counts in the control and AO‐1 groups were 2.1 and 1.6 log_10_ CFU/g on day 4, while coliforms were initially undetectable (<1 log_10_ CFU/g) in the AO‐5 and AO‐10 groups. Approximately 0.2, 0.5, and 0.8 log_10_ CFU/g reductions in coliforms compared with the control were observed in the AO‐1, AO‐5, and AO‐10 groups on day 12. Compared with the control group, AO‐5 and AO‐10 treatments significantly (*p* < .05) inhibited the growth of yeast during storage for 12 days (Figure 1c). Moreover, reductions in yeast counts of approximately 1.1–1.4 log_10_ CFU/g were achieved in AO‐5 and AO‐10 on day 12 compared with counts in the control. The molds of fresh‐cut cabbages could be detected in the control, AO‐1, and AO‐5 groups on day 8 but were not detected in AO‐10 during 12 days of storage. A significant (*p* < .05) reduction in mold was observed in the groups treated with aqueous ozone compared with the control group on day 12 (Figure 1d).

Furthermore, our results indicated that the initial disinfection efficiency of aqueous ozone is positively related to the treatment duration. AO‐10 reduced (*p* < .05) the counts of aerobic bacteria, coliforms, yeasts, and molds in fresh‐cut cabbage more substantially than AO‐1 and AO‐5. Ozone can attack multiple cellular components, including proteins, unsaturated lipids, and respiratory enzymes in cell membranes, peptidoglycans in cell envelopes, enzymes, and nucleic acids in the cytoplasm, and proteins and peptidoglycans in spore coats and viral capsids, and the bactericidal mechanism is therefore very complicated (Miller et al., 2013). In this experiment, aqueous ozone notably reduced aerobic bacteria, coliforms, yeasts, and molds on fresh‐cut cabbage. These results agreed with those of Papachristodoulou et al. (2018), who showed that aqueous ozone decreased Gram‐ and *Enterobacteriaceae* sp. loads on fresh‐cut melon during the first 5 days of storage. Sengun and Kendirci (2018) observed that aqueous ozone treatment could effectively reduce total mesothermal aerobic bacteria (TMAB), mold, and yeast in fresh‐cut lettuce and further found that aqueous ozone treatment at 4°C was particularly conducive to reducing TMAB counts, while aqueous ozone treatment at 15°C was more effective in reducing mold and yeast counts. In contrast, several studies of aqueous ozone using fresh‐cut fruits and vegetables have shown that reductions in microbial populations are low and equivalent to those of chlorination or even of a simple water wash. Beirão‐Da‐Costa, Moura‐Guedes, Ferreira‐Pinto, Empis, and Moldão‐Martins (2014) found that no decontamination effect of aqueous ozone (0.2 ppm 2 min^‐1^) on fresh‐cut kiwifruit. Differences in the disinfection effects of aqueous ozone on fresh‐cut fruits and vegetables may be related to the product type, initial microbial load, concentration of aqueous ozone, and treatment time (Liu et al., 2016).

### 3.2 Respiration rate and ethylene production analyses

**FIGURE 2:**
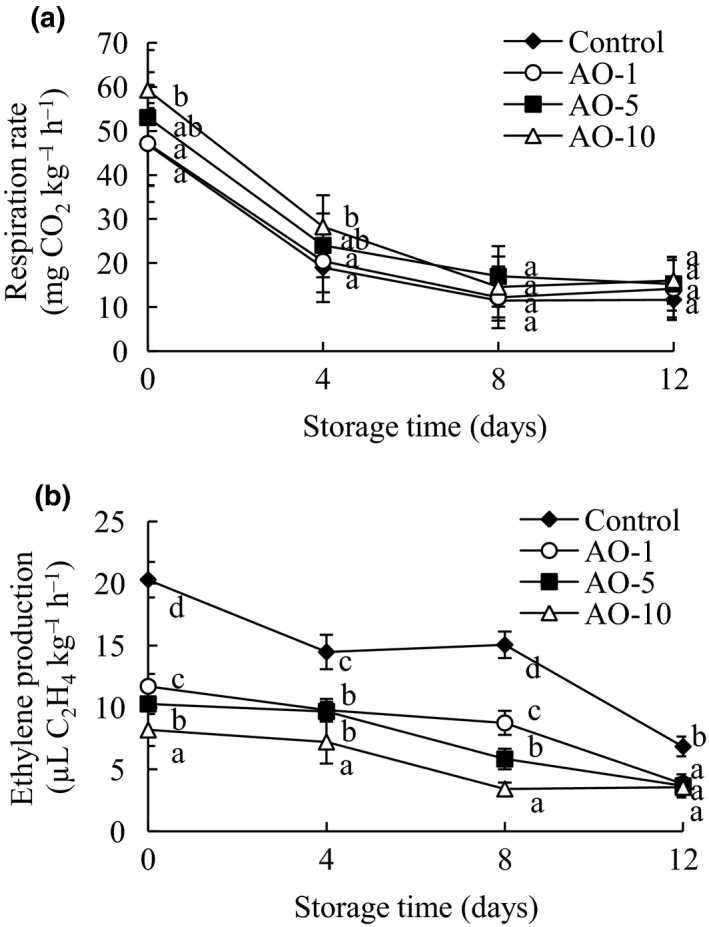
Effects of aqueous ozone treatment on respiration rates (A) and ethylene production (B) of fresh‐cut cabbages during 12 days of storage (◆, Control; ○, AO‐1; ■, AO‐5; △, AO‐10). Data shown are the means (*n* = 3) ± standard deviation. For each treatment, different lower letters show significantly different (*p *< .05) in the same storage days

Aqueous ozone treatment for 1, 5, and 10 min caused initial increases in the respiration rate of 0.24, 6.14, and 12.35 mg CO_2_ kg^‐1^ hr^‐1^ compared with that of the control group (Figure 2a). This result suggests that aqueous ozone provoked additional stress, causing some type of damage at the cellular level, which was expressed as an increase in respiratory activity (Silveira et al., 2018). This is consistent with previous reports showing that the initial respiration rates of tomato slices and minimally processed grape berries increased sharply after aqueous ozone treatment compared with that of the control (Aguayo et al., 2014; Silveira et al., 2018). With an increase in storage time, the respiratory rates of all groups decreased gradually, and there was no significant difference between groups at the later stages of storage. The effect of aqueous ozone on the respiration rate of fresh‐cut products is controversial (Chauhan, Raju, Ravi, Singh, & Bawa, 2011; Papachristodoulou et al., 2018). It is possible that the effect of ozone water on the respiration rate depends on several factors, such as the type of vegetal tissue, product variety, dose, treatment time, and temperature (Silveira et al., 2018).

Ethylene production was highest (*p* < .05) in all groups on day 0 of storage and then decreased gradually (Figure 2b). The ethylene production of samples treated with aqueous ozone was significantly (*p* < .05) lower than that of the control group during storage, and AO‐10 treatment had the greatest (*p* < .05) inhibitory effect on ethylene production. Aqueous ozone can reduce ethylene production in fresh‐cut cabbages, which may be due to the strong oxidation of ozone inactivating the related enzymes in ethylene synthesis, thereby blocking ethylene biosynthesis.

### 3.3 Quality attribute analyses

**FIGURE 3:**
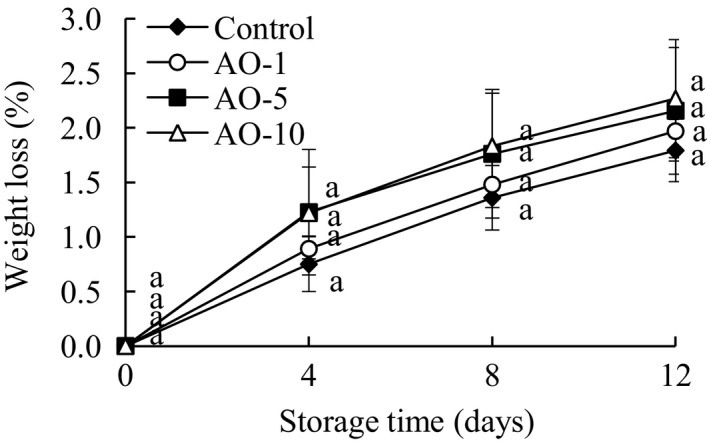
Effects of aqueous ozone treatment on weight loss of fresh‐cut cabbages during 12 days of storage (◆, Control; ○, AO‐1; ■, AO‐5; △, AO‐10). Data shown are the means (*n* = 3) ± standard deviation. For each treatment, different lower letters show significantly different (*p *< .05) in the same storage days

As shown in Figure 3, the weight loss in all groups increased markedly (*p* < .05) as storage time increased. No differences in the weight loss of fresh‐cut cabbages treated with control and aqueous ozone were observed. However, it was found that a slight increase in weight loss in fresh‐cut cabbages treated with aqueous ozone, depending on treat time. The weight loss of fresh‐cut cabbages during storage may be attributable to microbial growth, changes in physiological metabolism, and moisture transpiration. The microbial growth can accelerate weight loss, while aqueous ozone can inhibit the microbial growth to slow down it. However, the observed increase in weight loss may be enhanced by the strong oxidation of aqueous ozone, which causes increased membrane damage of cabbage to accelerate moisture transpiration. Enhanced respiratory metabolism also consumes more nutrients, resulting in accelerated weight loss.

**FIGURE 4:**
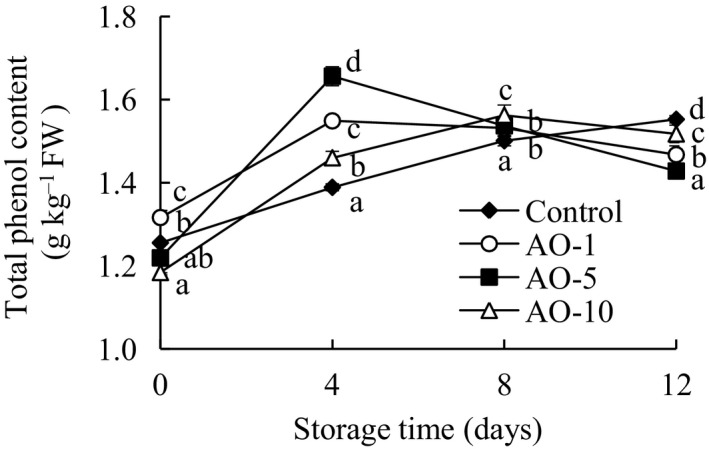
Effects of aqueous ozone treatment on total phenol content of fresh‐cut cabbages during 12 days of storage (◆, Control; ○, AO‐1; ■, AO‐5; △, AO‐10). Data shown are the means (*n* = 3) ± standard deviation. For each treatment, different lower letters show significantly different (*p *< .05) in the same storage days

Phenols represent a significant part of the total antioxidant content in fruits and vegetables. According to the results shown in Figure 4, the total phenol content of samples treated with aqueous ozone was significantly (*p* < .05) higher than that of the control group on days 4 and 8 of storage, and the AO‐5 group exhibited the highest (*p* < .05) accumulation of phenols on day 4 of storage. However, the total phenol content of aqueous ozone treatment groups was notably (*p* < .05) lower than that of the control group on day 12 of storage. At present, the published results on the effect of aqueous ozone on total phenol content are contradictory. Silveira et al. (2018) and Alothman et al. (2010) inferred that aqueous ozone treatments increased polyphenol content, which was due to the defensive response of stress‐affected cells to ozone stimulation, resulting in a rapid increase in enzymes in the phenylpropanol pathway to synthesize polyphenols. In contradictory results, several studies showed that aqueous ozone caused significant degradation of phenolic compounds in fresh‐cut fruits and vegetables (Chauhan et al., 2011; Liu et al., 2016). The degradation of polyphenols may, therefore, be the result of several possible chemical reactions involving ozone.

**FIGURE 5:**
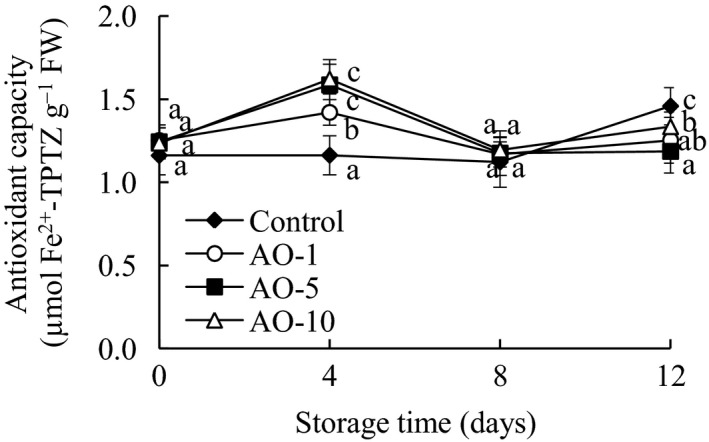
Effects of aqueous ozone treatment on antioxidant capacity of fresh‐cut cabbages during 12 days of storage (◆, Control; ○, AO‐1; ■, AO‐5; △, AO‐10). Data shown are the means (*n* = 3) ± standard deviation. For each treatment, different lower letters show significantly different (*p *< .05) in the same storage days

Vegetables are an important part of a healthy diet, and the principal source of natural antioxidants. As shown in Figure 5, the antioxidant capacity of the aqueous ozone treatment groups was significantly (*p* < .05) higher than that of the control group on day 4 of storage, but their antioxidant capacity was markedly (*p* < .05) lower that of the control group on day 12. This trend was similar to that of total phenol content changes. Besides being rich in polyphenols, cabbage also contains antioxidant phytochemicals such as glucosinolates, carotene, tocopherol, and ascobate, which can also affect the antioxidant capacity of cabbage (Oboh et al., 2017).

**FIGURE 6:**
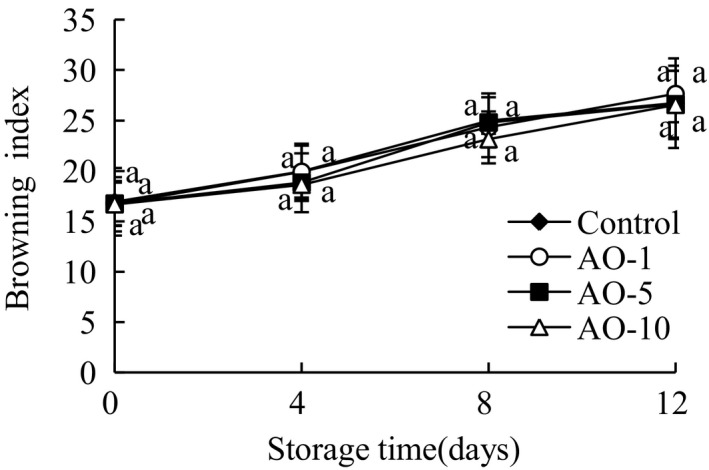
Effects of aqueous ozone treatment on browning index of fresh‐cut cabbages during 12 days of storage (◆, Control; ○, AO‐1; ■, AO‐5; △, AO‐10). Data shown are the means (*n* = 3) ± standard deviation. For each treatment, different lower letters show significantly different (*p *< .05) in the same storage days

The changes in BI of fresh‐cut cabbages are shown in Figure 6. The browning degree of all groups increased gradually during storage, and their BI increased observably (*p* < .05) during the 4–8 days of storage. It was observed that, the aqueous ozone treated samples had lesser BI values, as compared to the control samples. No significant differences were observed, indicatings that aqueous ozone treatments did not affect the color of the samples adversely and the results obtained were similar to the studies done by Ummat, Singh, and Sidhu (2018), on shredded green bell pepper, that stated that aqueous ozone had no effect on color. Also Wang et al. (2019) found that fresh‐cut lettuce treated with aqueous ozone showed no effect on the color.

**FIGURE 7:**
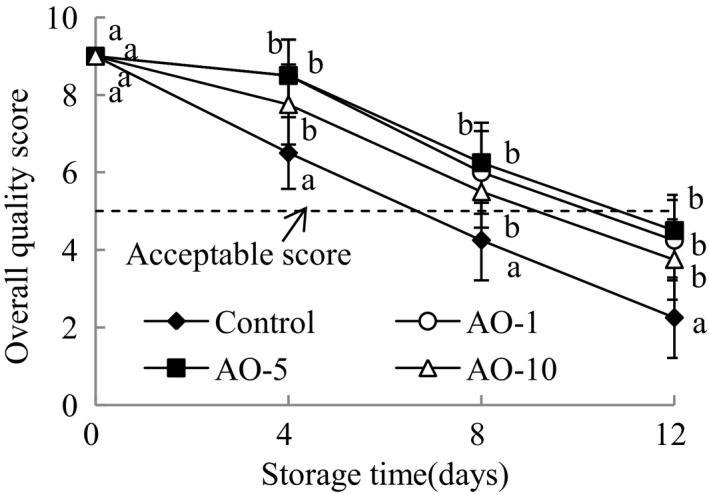
Effects of aqueous ozone treatment on overall quality of fresh‐cut cabbages during 12 days of storage (◆, Control; ○, AO‐1; ■, AO‐5; △, AO‐10). Data shown are the means (*n* = 3) ± standard deviation. For each treatment, different lower letters show significantly different (*p *< .05) in the same storage days

Sensorial results for the overall quality of fresh‐cut cabbage are summarized in Figure 7. The overall sensory score for the control group was below the acceptable limit for marketability on day 8 of storage, while those for the aqueous ozone treatment groups were below the acceptable limit on day 12. There were no significant differences among aqueous ozone treatment groups in overall quality scores during storage. The aqueous ozone treatments delayed the decline in the overall quality of fresh‐cut cabbage, and AO‐5 had the best preservation effect. Some researchers have found the positive effects of aqueous ozone in the maintenance of sensory quality of fresh‐cut fruits and vegetables such as carrot and “Galia” melon during storage (Chauhan et al., 2011; Silveira, Aguayo, & Artés, 2010). These results differ from that observed when tomato slices were treated with aqueous ozone. Aguayo et al. (2014) found that the changes in sensorial parameters of tomato slices were only influenced by time of storage, but not by aqueous ozone treatment.

The process conditions of aqueous ozone must prevent, as far as possible, excessive losses of quality attributes. A comprehensive analysis showed that AO‐5 maintained better quality attributes of fresh‐cut cabbages.

### 3.4 Residual pesticide analyses

**FIGURE 8:**
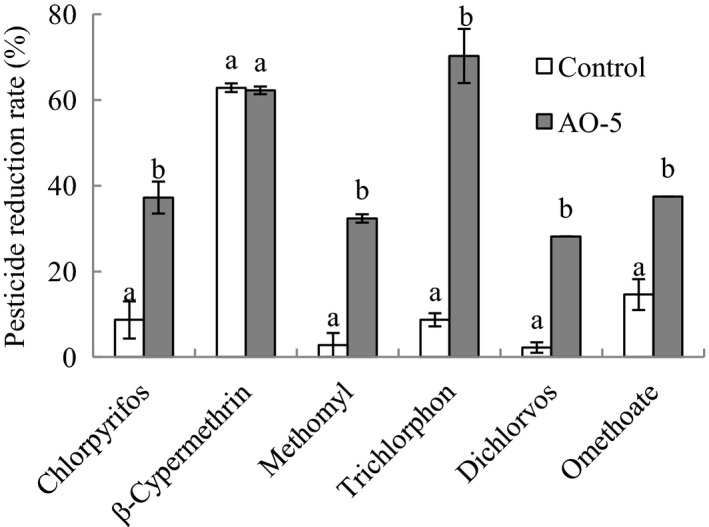
Effects of aqueous ozone treatment on removal of chlorpyrifos, β‐cypermethrin, trichlorfon, methomyl, dichlorvos, and omethoate residues in fresh‐cut cabbages (, Control;, AO‐5). Data shown are the means (*n* = 3) ± standard deviation. For each treatment, different lower letters show significantly different (*p *< .05) in the same storage days

Recent studies have confirmed that aqueous ozone washing is a safe and effective treatment for the removal of pesticide residues to improve consumer health (Guo & Wang, 2017). In the "Rules for the Implementation of Sample Inspection of National Food Safety Supervision" (2019) formulated by the general administration of market supervision of China, it is stipulated that 19 kinds of pesticides residues must be detected in cabbages, and their maximum residues are limited in GB2763. In this study, 6 kinds of pesticides commonly used in the planting process of cabbage were taken as the research objective, including chlorpyrifos, 1β‐cypermethrin, trichlorfon, methomyl, dichlorvos, and omethoate. The effect of aqueous ozone treatment on six pesticide residues in fresh‐cut cabbage is shown in Figure 8. Except for β‐cypermethrin, the removal efficiency of aqueous ozone treatment on the pesticide residues was significantly (*p* < .05) higher than that of the control treatment. The maximum removal of trichlorfon (70.30%) from fresh‐cut cabbage after treatment with AO‐5 was about 8 times greater (*p* < .05) than that of water treatment for 5 min. The removal efficiencies of chlorpyrifos, methomyl, dichlorvos, and omethoate in the AO‐5 group were 37.26%, 32.37%, 28.13%, and 37.50%, while those in the control group were only 8.70%, 2.80%, 2.21%, and 14.58%. In addition, both AO‐5 and control treatments effectively removed the residues of β‐cypermethrin, with removal efficiencies of 62.28% and 62.87%, respectively. Accordingly, analytical results demonstrated that aqueous ozone treatment is an effective method to remove pesticide residues.

Ozone undergoes oxidation–reduction reactions in water to produce monatomic oxygen (O), hydroxyl (OH^‐^), and hydroxyl radicals (OH·), which have strong oxidation capabilities. Therefore, aqueous ozone can break the strong polar bonds in organic pesticide molecules to form small molecular compounds, such as acids, alcohols, amines, or their oxides. Several studies have evaluated the removal of pesticide residues by aqueous ozone. In a study of strawberries, Lozowicka et al. (2016) showed that the efficacy of aqueous ozone was greater (*p* < .05) than that of tap water. Both ozone gas and ozone water effectively removed pesticide residues from harvested carrots without forming toxic intermediates, and their removal efficiencies increased with increases in ozone concentration and treatment time (Souza et al., 2018). However, in fresh‐cut fruits and vegetables, long washing periods are not suitable because they can lead to the loss of cell fluids and the penetration of the washing solution from wounds into cells, so this experiment only considered the effect of AO‐5 treatment on the removal of pesticide residues. In addition, the by‐products produced by aqueous ozone degradation pesticides may be more toxic than the pesticides themselves, which may be a potential risk and worthy of further research.

## 4 CONCLUSIONS

The results indicated that aqueous ozone notably reduced microbial counts, and AO‐10 treatment had the best inhibitory effect on the growth of aerobic bacteria, coliforms, and yeasts during storage. Although aqueous ozone stimulated initial respiratory metabolism of fresh‐cut cabbage, it significantly inhibited ethylene production during 12 days of storage, delayed the decline in sensory qualities, and extended the shelf life to 8 days. Moreover, aqueous ozone treatment effectively removed six pesticide residues from fresh‐cut cabbage, and the removal efficiencies for trichlorfon, chlorpyrifos, methomyl, dichlorvos, and omethoate were significantly (*p* < .05) higher than those of the control treatment. For the industrial production of fresh‐cut fruits and vegetables, the treatment time must be shortened to improve efficiency; accordingly, aqueous ozone treatment for 5 min is particularly suitable for fresh‐cut cabbage as a washing technology.

## CONFLICT OF INTEREST

The authors of this paper declare no conflict of interest.

## ETHICAL STATEMENTS

This study does not involve any human or animal testings.
